# Integrated analysis of the microbiota-gut-brain axis in response to sleep deprivation and diet-induced obesity

**DOI:** 10.3389/fendo.2023.1117259

**Published:** 2023-02-21

**Authors:** Jibeom Lee, Jiseung Kang, Yumin Kim, Sunjae Lee, Chang-Myung Oh, Tae Kim

**Affiliations:** ^1^ Department of Biomedical Science and Engineering, Gwangju Institute of Science and Technology, Gwangju, Republic of Korea; ^2^ Department of School of Life Sciences, Gwangju Institute of Science and Technology, Gwangju, Republic of Korea

**Keywords:** gut microbiota, gut-brain axis, sleep deprivation, diet induced obesity, bioinformatics

## Abstract

**Introduction:**

Sleep deprivation (SD) and obesity are common in modern societies. SD and obesity frequently coexist, but research on the combined consequences of SD and obesity has been limited. In this study, we investigated the gut microbiota and host responses to SD and high-fat diet (HFD)-induced obesity. In addition, we attempted to identify key mediators of the microbiota-gut-brain axis.

**Methods:**

C57BL/6J mice were divided into four groups based on whether they were sleep deprived and whether they were fed a standard chow diet (SCD) or HFD. We then performed fecal microbiome shotgun sequencing, gut transcriptome analysis using RNA sequencing, and brain mRNA expression analysis using the nanoString nCounter Mouse Neuroinflammation Panel.

**Results:**

The gut microbiota was significantly altered by the HFD, whereas the gut transcriptome was primarily influenced by SD. Sleep and diet are both important in the inflammatory system of the brain. When SD and the HFD were combined, the inflammatory system of the brain was severely disrupted. In addition, inosine-5' phosphate may be the gut microbial metabolite that mediates microbiota-gut-brain interactions. To identify the major drivers of this interaction, we analyzed the multi-omics data. Integrative analysis revealed two driver factors that were mostly composed of the gut microbiota. We discovered that the gut microbiota may be the primary driver of microbiota-gut-brain interactions.

**Discussion:**

These findings imply that healing gut dysbiosis may be a viable therapeutic target for enhancing sleep quality and curing obesity-related dysfunction.

## 1 Introduction

Sleep deprivation (SD) and obesity are common in modern society. In the United States, only 65 percent of individuals report sleeping for 7 hours or more per day. The average prevalence of obesity in adults was 19.5 percent across OECD nations in 2015 (1). These two clinical conditions are now recognized as serious problems, because sleep and diet are both important for maintaining physical and mental health. Insufficient sleep leads to metabolic imbalance and an increased risk of metabolic diseases, including cardiovascular disease (CVD), type 2 diabetes mellitus (T2DM), hypertension, obesity, and depression (2, 3). Even acute SD can cause altered glucose metabolism, changes in hormone production, and weight gain (4–6). Similarly, obesity has been linked to a variety of modern diseases, including T2DM, CVD, and other metabolic illnesses (7).

Over the past decade, the microbiome has emerged as a significant component of human health (8). Many studies have revealed that the gut microbiome communicates with the host and participates in the regulation of the systemic immune, metabolic, and nervous systems, as well as gut metabolism (9). The gut-brain axis (GBA) is a bidirectional communication pathway between the gut and the brain. Recent research has revealed that gut microbiota may play a role in mediating these interactions, which have been dubbed microbiota-gut-brain interactions (9, 10).

Diet and obesity are well-recognized contributors to the gut microbiome (11). Obesity is a major risk factor for gut dysbiosis, and the gut microbiota contribute to metabolic dysfunction in obese individuals (12). SD also induces dysbiosis in the gut microbiome. Sleep disturbance and loss are linked to systemic inflammation and oxidative stress in the gut (13, 14). Insomnia leads to significant structural and functional changes in the gut microbiome (15), which fluctuates in response to disturbances in sleep and circadian rhythm (16). The gut microbiota can also influence sleep quality through the GBA (17).

In modern society, there are concurrent epidemics of SD and obesity with a potential bidirectional relationship (18). While SD is a well-known risk factor for obesity (19), obesity can also cause sleep disorders, such as insomnia, obstructive sleep apnea, and obesity hypoventilation syndrome (20). Although SD and obesity frequently coexist, research on the combined consequences of SD and obesity have been limited. Furthermore, to the best of our knowledge, there have been no studies on the key regulators of microbiota-gut-brain interactions in the gut and brain. In this study, we investigated the shotgun metagenomic sequencing and host gene expression profiles in response to SD and obesity in a mouse model. In addition, we performed a multi-omics factor analysis to identify a major driver of these profile alterations in response to metabolic stress caused by SD and obesity.

## 2 Materials and methods

### 2.1 Mouse experimental model

**Figure 1:**
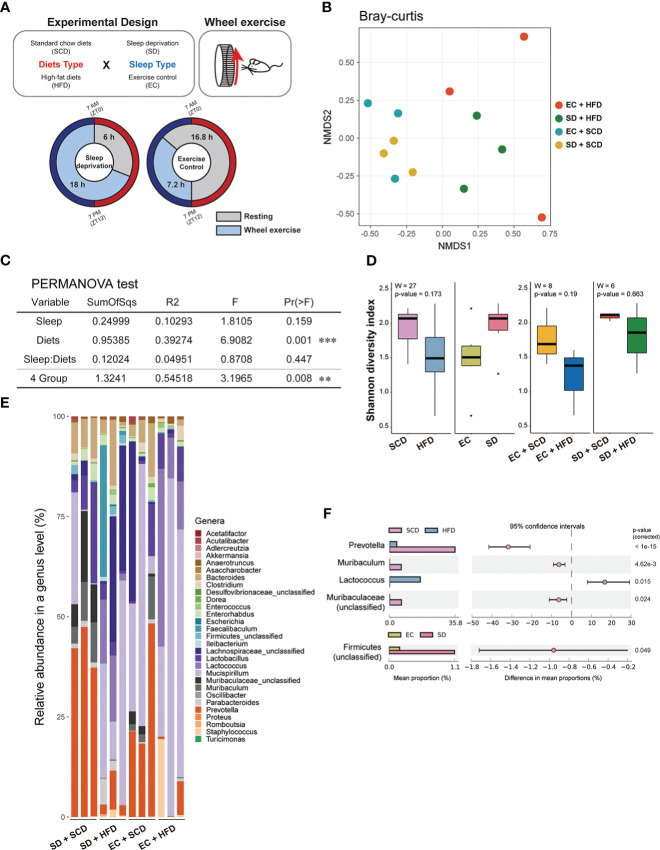
Gut flora depending on sleep conditions and diet type. The experimental design is described graphically here by the following two factors: diet type and sleep **(A)**. The sample distance for β-diversity was calculated using the Bray-Curtis method **(B)**. Permutational multivariate analysis of variance was applied to compare the means of samples for a single factor, either diet or sleep type, and two factors **(C)**. The α-diversity was measured using the Shannon index, and the median was analyzed using the Wilcoxon test, depending on two factors (left) and specifically diets for each sleep condition (right) **(D)**. The relative abundance of 29 genera in the 12 samples indicated a different proposition at the genus level **(E)**. The mean proportion and 95% confidence interval (*P* < 0.05) explained the main genus contributors related to each factor **(F)**. *P < 0.05, **P < 0.01 and ***P < 0.001

Eight-week-old C57BL6/J male mice were randomly divided into four cages and fed one of two diets: a standard chow diet or high-fat diet (D12492; Research Diets, Inc., New Brunswick, NJ, USA). Sleep conditions were either SD or exercise control (EC) after eight weeks, with one day of habituation inside the wheel and five days in the sleep environment, as stated in the graphical abstract (**Figure 1A**; **Supplementary Figure 1**). C57BL/6J mice were then divided into four groups based on whether they were deprived of sleep and whether they were fed a standard chow diet (SCD) or high-fat diet (HFD): EC+SCD, EC+HFD, SD+SCD, and SD+HFD (number of mice per group = 3, the minimum requirement for statistics). These mice were fed SD or HFD for 8 weeks as previously described (21, 22). All mice were maintained in a 12 h dark-light cycle, with the lights turning on at 7 AM and off at 7 PM. There are no inclusion/exclusion criteria for mice selection for the analysis. Because C57BL/6J mouse is genetically homogeneous inbred strain.

### 2.2 Mouse sleep deprivation

SD was achieved by placing mice individually in activity wheels (Lafeyette Instruments, Lafayette, IN, USA) that had a motorized wheel with a diameter of 6.985 cm and an internal wheel with a diameter of 5.715 cm, with free access to food and water, as previously described (23). First, the mice were habituated to the activity wheel conditions for 24 h. We conducted experiments on the SD mice, including the SD+SCD and SD+HFD groups, over the course of five days using a slow rotational movement of the activity wheel (programmed on a schedule of 3 s ‘on’ and 12 s ‘off’) for 18 h (from ZT 06 to ZT 24) as previously described (24). SD in this study involved forcing the mice to move to interrupt their sleep. Exercise control is necessary to avoid confusing the interpretation of experimental results because of the nonspecific effects of movement itself. For five days, mice were provided with the same walking distance (EC+SCD and EC+HFD groups) but with a wheel on/off schedule of 15 min on/15 min off at 3 m/min speed for 7.2 h, enabling them to sleep uninterrupted for longer periods.

### 2.3 Tissue harvest and RNA precipitation

Mouse tissues were all harvested at 7 AM, and the harvest time in each mouse sample did not exceed 3 min, limiting variations and stress environments between the groups. After cervical dislocation, whole blood was collected from the left ventricle of the heart tissue and perfused with 4% paraformaldehyde (PFA). Whole blood was centrifuged for 10 min at 6000 rpm, and floating serum was collected in a clean container. Blood sera were collected and stored at -80°C. Overnight storage of brain tissue was performed in a 4% PFA solution.

RNA from colon samples was extracted using TRIzol (Invitrogen, Waltham, MA, USA) and followed up according to the manufacturer’s protocol. The extracted RNA was then stored at < -80°C. The brain was divided vertically, and the right hemisphere was used for RNA extraction with a PureLink FFPE RNA Isolation Kit (Invitrogen).

### 2.4 Colon RNA sequencing

The quality of RNA from colon tissue was checked using the Agilent 2100 bioanalyzer. Only samples with a high-quality score (RNA Integrity Number) of 6 or higher were used for making a library. The quality of the sequencing was also checked using a Phred quality score (Q score). More than 92% of the sequencing had a high score of Q30, which means the accuracy of the sequencing was at 99.9%. Colon RNA FASTQ was automatically counted as the transcript amount using Kallisto (version 0.45.0). DESeq2 packages were used to extract the list of differentially expressed genes (DEGs) with a design matrix for diet, sleep, and interaction terms. Volcanoplot was used to compare DEG analysis results without interaction terms, and with an adjusted *P*-value cutoff of 0.01 and absolute value of log2 transformed fold-change 2. For pathway enrichment analysis using clusterprofiler packages (version 4.0.5), such as Gene Ontology (GO) and Kyoto Encyclopedia of Genes and Genomes (KEGG) database-based pathway enrichment, the input list had significant values that were below the adjusted *P*-value of 0.05 for the DEseq2 results. Input data were based on the Entrez ID labeling, which indicates that non-available gene names in the Entrez ID are automatically removed. During the analysis, the number of genes enriched in KEGG function was 499 and 557 in SD *vs*. EC (Ref. SCD) and SD *vs*. EC (Ref. HFD), respectively; however, the filtered genes were automatically recognized at 479 and 541, respectively. The results of the pathway enrichment analysis based on GO biological processes were 199 and 371 in SD *vs*. EC (Ref. SCD) and SD *vs*. EC (Ref. HFD), respectively. Gene set enrichment analysis (GSEA) was performed using the GSEA software (version 4.1.0) on 3895 gene sets. The enrichment map of GSEA was programmed in Cytoscape (version 3.8.2) using the enrichmentMap tool (version 3.3.3).

### 2.5 Shotgun metagenomic sequencing

Using sterilized forceps, internal feces from the colon were collected, and colon tissue was washed with phosphate-buffered saline to remove any remaining feces and then stored at -80°C. Shotgun sequencing was used to sequence precipitated fecal DNA. The total number of reads produced from 12 samples averaged 76,004,118 ± 636,745.9436, with at least 92% of the nucleotides having a Phred quality score ≥30. Overall, a total of 6 different phyla, 22 families, 29 genera, and 53 species were detected using Metaphlan (version 3.0). The HUMAnN program (version 3.0) was used to measured estimated genes, as referred by uniref90, which were quantified as copies per million, and the total sum of all genes ranged from 1,294,101 to 1,467,302, depending on the samples. STAMP (version 2.1.3) and Mofapy2 (version 1.2.2) were used to examine the metagenomic data from the relative abundance results (version 0.6.4). α- and β-diversities were calculated from species-level taxa using the vegan package (version 2.5-7). In particular, for Mofapy2, significant gene sets of positive and negative weights from colon RNA sequencing and brain nCounter were manually extracted from the GO database.

### 2.6 Brain nanoString nCounter

Brain RNA samples were loaded onto the nCounter Inflammation Panel (nanoString, Seattle, WA, USA). The cartridge counted the probe-tagged fluorescent barcode with a digital analyzer equipped with a microscope. One molecule was quantified as the count value. Gene expression levels between samples were confirmed using 13 housekeeping genes: *Aars, Asb10, Ccdc127, Cnot10, Csnk2a2, Fam104a, Gusb, Lars, Mto1, Supt7l, Tada2b, Tbp*, and *Spnpep1*. Counting data from the nanoString nCounter analysis were analyzed using R software (versions 4.1.0 and 4.1.1). The DEseq2 package was used to analyze the DEGs.

### 2.7 Ethical statement

All experiments were reviewed and approved by the Institutional Animal Care and Use Committee of Gwangju Institute of Science and Technology (Approval number: GIST-2021-064).

## 3 Results

### 3.1 High-fat diet mainly loaded gut microbiome diversity

**Figure 2:**
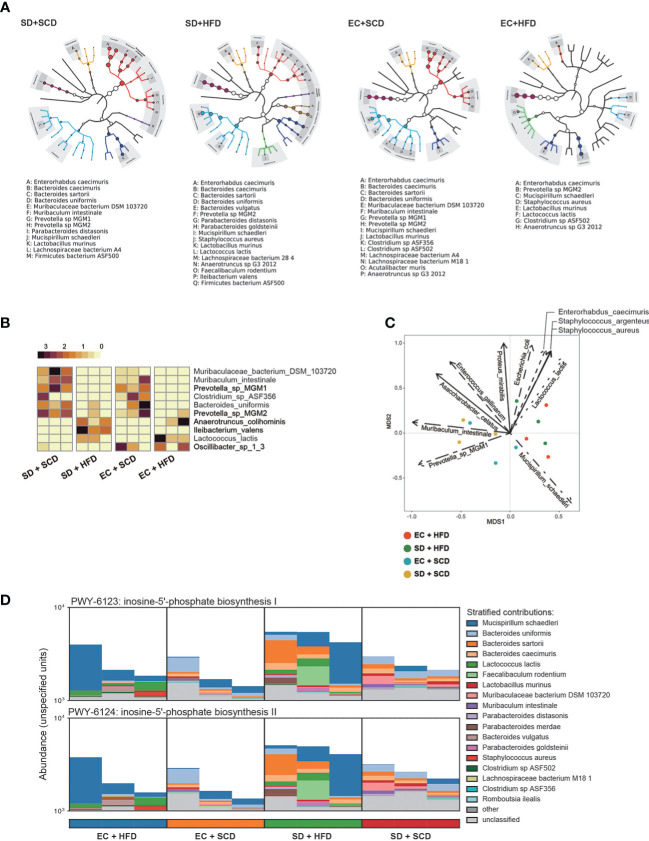
Difference for genetic expression and clade distribution of the gut microbiome. The clade distribution pattern in a group (n=3) is referred to as a tree cladogram **(A)**. The top 10 significantly variable gut microbiota species were measured using an analysis of variance test depending on sleep and diet conditions (*P* < 0.05) and expressed as z-scores of relative abundance values **(B)**. Eleven environmental factors affecting Bray–Curtis β-diversity and its ordination are shown as arrows (*P* < 0.005) **(C)**. The inosine-5’-phosphate biosynthesis I and II were estimated from DNA reads, and species attribution was identified **(D)**.

To determine the impact of SD and HFD-induced obesity on the baseline composition of the gut microbiome, fecal samples were collected, and shotgun metagenome sequencing was performed for taxonomic profiling and functional analysis. To calculate inter-group dissimilarity (beta-diversity), we computed the Bray-Curtis dissimilarity, and unweighted and weighted Unifrac. Beta-diversity-based principal coordinate analysis plots (**Figure 1B**; **Supplementary Figure 2**) showed a strong distinction by diet. This means that diets had a greater impact on gut microbial diversity than that of sleep variables. In the permutational multivariate analysis of variance test (**Figure 2C**), diet showed a significant effect, but sleep did not cause significant differences between the groups (**Figure 1C**).

At the phylum level, the *Firmicutes/Bacteroidetes* (F/B) ratio is significantly associated with intestinal homeostasis (25). The HFD significantly increased the microbiota F/B ratio by increasing the content of *Firmicutes*; in contrast, SD decreased the F/B ratio, but the difference was not statistically significant (**Tables 1**, **2**). **Figure 1D** shows the intragroup diversity (alpha diversity) expressed using the Shannon index. The HFD decreased alpha diversity while SD increased alpha diversity, but these changes were not statistically significant. At the genus level, the HFD decreased the relative abundance of the genera *Prevotella* and *Muribaculum* and increased that of *Lactococcus*. SD increased the abundance of *Firmicutes* (**Figures 1E, F**).

**Table 1:** The significant taxa either diets or sleep parameter.

	PARAMETERS	TAXA	W	p-value (< 0.05)
***PHYLA***	Diets	*Bacteroidetes*	1	0.008
		*Firmicutes*	30	0.066
		*F/B*	36	0.005
***FAMILIES***	Diets	*Muribaculaceae*	0	0.005
		*Prevotellaceae*	0	0.005
		*Staphylococcaceae*	34	0.013
		*Enterococcaceae*	32	0.025
		*Streptococcaceae*	36	0.003
	Sleep	*Tannerellaceae*	3	0.020
***GENERA***	Diets	*Muribaculaceae (unclassified)*	0	0.005
		*Muribaculum*	0	0.004
		*Prevotella*	0	0.005
		*Staphylococcus*	34	0.013
		*Enterococcus*	32	0.025
		*Lactococcus*	36	0.003
	Sleep	*Parabacteroides*	3	0.020
***SPECIES***	Diets	*Bacteroides caecimuris*	5	0.045
		*Bacteroides uniformis*	2	0.013
		*Muribaculaceae bacterium DSM 103720*	0	0.005
		*Muribaculum intestinale*	0	0.004
		*Prevotella sp MGM1*	0	0.003
		*Prevotella sp MGM2*	4	0.031
		*Staphylococcus aureus*	34	0.013
		*Enterococcus gallinarum*	32	0.025
		*Lactococcus lactis*	36	0.003
		*Clostridium sp ASF356*	0	0.003
		*Lachnospiraceae bacterium M18 1*	2	0.013
		*Anaerotruncus colihominis*	33	0.010
	Sleep	*Bacteroides sartorii*	3	0.020
		*Parabacteroides distasonis*	5	0.045
		*Parabacteroides merdae*	4.5	0.026

In 4 taxonomy classification including phyla, familes, genera, species, significant taxa were measured as wilcoxon test depending on the variable, diets and sleep. The total number of each taxanomy were 7 phyla, 22 famila, 29 genera and 53 species as metaphlan results. This table only described the p-value below 0.05.

**Table 2:** Firmicutes, bacteroidetes and both ratios.

Group	n	Firmicutes		Bacteroidetes		F/B	
		Median	IQR	Median	IQR	Median	IQR
SD + SCD	3	13.09	11.74-20.24	67.95	64.37-75.73	0.17	0.16-0.29
SD + HFD	3	56.23	47.96-57.47	12.47	8.09-20.62	4.51	3.28-5.75
EC + SCD	3	18.24	11.91-30.63	29.28	28.81-51.63	0.25	0.22-0.86
EC + HFD	3	25.94	20.61-50.57	0.87	0.56-5.89	62.01	32.20-74.21

The median and interquartile range (IQR) of firmicutes, bacteroidetes, and those ratios were specifically explained in the table. There were 3 in each category. SD, sleep deprivation; SCD, standard chow diet; HFD, high fat diet; EC, exercise control.

### 3.2 Predictive metabolomic profiling of gut microbiota following sleep deprivation and consumption of a high-fat diet

Gut microbiota composition was then examined at the species level. **Figure 2A** shows the diverse compositions of the bacterial species within each group. The HFD reduced the relative abundance of *Muribaculum intestinale* and *Prevotella* MGM1 and MGM2 and increased the abundance of *Anaerotruncus colihominis* and *Lactococcus lactis*. When compared to that in the control (EC+SCD group), Muribaculaceae *bacteria* DSM 103720 abundance increased in the SD+SCD group but not in the SD+HFD group. When SD and an HFD were combined, *Ileibacterium valens* was a key player (**Figure 2B**). Principal component analysis (PCA) ordination plots of the relative abundance of species indicated the main drivers of each principal component (**Figure 2C**).

The gut microbiota is an important part of host digestion, and this process results in hundreds of microbial metabolites (26). Recent findings suggest that there is a bidirectional link between the brain and intestine, the so-called GBA; microbial metabolites are major mediators of this communication (26–28). To understand the metabolic effects of sleep and diet, we performed strain-level functional pathway-enriched pathway analysis (**Figure 2D**). Among the enriched pathways, inosin-5’-phosphate (5’-IMP) biosynthesis-related strains were significantly increased in the SD+HFD group compared to those in the other groups.

### 3.3 Transcriptome analysis of mouse large intestine after sleep deprivation and consumption of a high-fat diet

**Figure 3:**
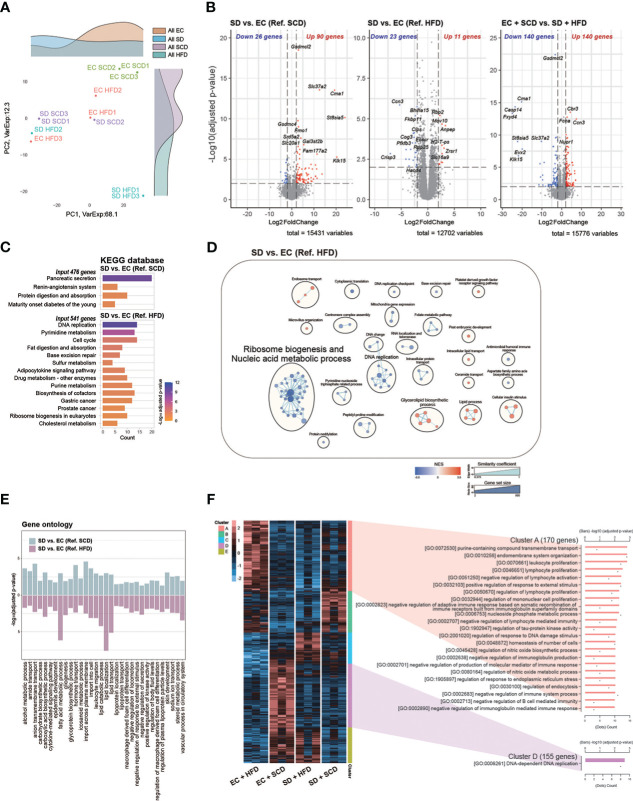
Colon RNA sequencing. Principal coordinate analysis plot **(A)**. Volcanoplot of three differentially expressed genes (DEGs) with cut-off conditions of adjusted *P*-value < 0.01 and |Log_2_ (fold change)| > 2 **(B)**. Kyoto Encyclopedia of Genes and Genomes enrichment analysis was performed for a group of genes (adjusted *P*-value < 0.05 and |Log_2_ (fold change)| > 1) **(C)**. Gene set enrichment analysis based on Gene ontology (GO) biological processes was plotted as an enrichment map; a total of 2487 and 1408 gene sets were upregulated in the sleep deprivation (SD) + high-fat diet (HFD) and exercise control (EC) + HFD groups, respectively **(D)**. The intersected 32 GO pathways between the two DEGs are described similarly **(E)**. In the case of normalized count levels, 613 genes were significantly different depending on the diet and sleep conditions, and two clusters were summarized in the GO BP pathway enrichment analysis **(F)**.

Next, to compare the effects of sleep and diet on the gut transcriptome, we performed RNA-seq analysis of the large intestine of mice from the EC+SCD, EC+HFD, SD+SCD, and SD+HFD groups. PCA was performed to reveal the major stress on the gut transcriptome between sleep and diet. **Figure 3A** depicts the results. Unlike the gut microbiota, which was mostly affected by diet, the gut transcriptome was primarily affected by sleep. In a DEG analysis (**Figure 3B**), 90 genes were upregulated, and 26 genes were downregulated in the SD+SCD group compared to those in the EC+SCD group. Gasdermin C-like 2 (*Gsdmcl2*), chymase 1 (*Cma1*), solute carrier family 37 member 2 (*Slc37a2*), Alpha-2,8-sialyltransferase 8E (*St8sia5*), and gasdermin C4 (*Gsdmc4*) genes were the top five upregulated DEGs (based on *P*-value). Stress-associated endoplasmic reticulum protein 1 (*Serp1*), death-associated protein 1(*Dap*), transmembrane protein 35A (*Tmem35a*), ubiquitin-like modifier enzyme 5 (*Uba5*), and bone gamma-carboxyglutamate protein 3 (*Bglap3*) were the top five downregulated DEGs (based on *P*-value). In the HFD group, only 11 genes were upregulated, and 23 genes were downregulated (SD+HFD *vs*. EC+HFD). The *Serp1* gene was also one of the top five downregulated genes in the SD+HFD group compared to that in the EC+HFD group (**Figure 3B**).

*Gsdmcl2, Cma1, Slc37a2*, and *St8sia5* were downregulated in the SD+HFD group compared to those in the EC+SCD group but were upregulated in the SD+SCD group compared to those in the EC+SCD group (**Figure 3B**). After SD, the tumor protein D52-like 1 (*Tpd52l1*), cellular communication network factor 3 (*Ccn3*), and UDP-GlcNAc:betaGal beta-1,3-N-acetylglucosaminyltransfera 9 (*B3gnt9*) genes were downregulated in both SCD- and HFD-fed mice (**Supplementary Figure 3**).

The KEGG enrichment analysis indicated that DEGs between the SD+SCD and EC+SCD groups were enriched in digestive system-related pathways, including “Pancreatic secretion” and “Protein digestion and absorption” (**Figure 3C**). The DEGs between the SD+HFD and EC+HFD groups were enriched in nucleic acid metabolism-related and lipid metabolism-related pathways (**Figures 3C, D**). **Figure 3E** shows the common GO terms related to SD. The nutrient metabolism-related terms, including “carbohydrate biosynthetic process,” “fatty acid metabolic process,” and “glycoprotein biosynthetic process,” and immune system-related terms, including “cytokine-mediated signaling pathway,” “leukocyte migration,” and “macrophage-derived foam cell differentiation,” were highly enriched in both the SCD and HFD conditions after SD (**Figure 3E**).

Using DEGs among the four groups, we performed heatmap clustering analysis. **Figure 3F** shows a heatmap of GO terms based on the DEGs in each cluster. Cluster A, which was composed of DEGs in the EC+HFD group, included immune system-related GO terms such as “leukocyte proliferation,” “lymphocyte proliferation,” and “regulation of mononuclear cell proliferation.” Cluster D, which was composed of DEGs in the EC+SCD group, included DNA replication-related genes (**Figure 3F**).

### 3.4 Neuroinflammatory changes in the brain after sleep deprivation and consumption of a high-fat diet

**Figure 4:**
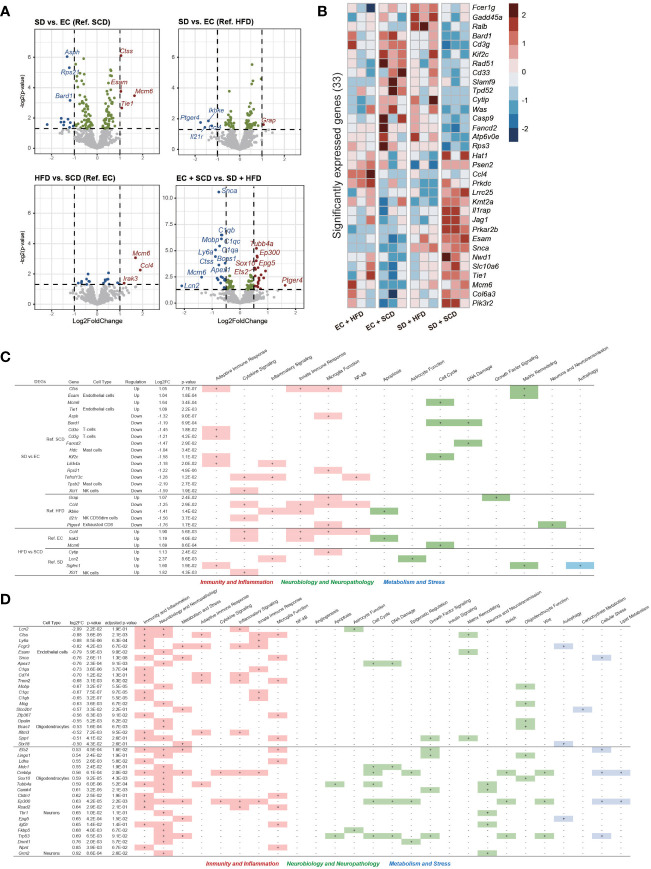
Brain Nanostring nCount analysis. Volcanoplot of the four differentially expressed gene (DEG) results (*P*-value < 0.05) **(A)**. A total of 33 significantly expressed genes are listed by z-score in the heatmap **(B)**. The table includes details regarding four DEGs in sleep deprivation (SD) + standard chow diet (SCD) *vs*. exercise control (EC) + SCD, SD + high-fat diet (HFD) *vs*. EC + HFD, EC + HFD *vs*. EC + SCD, and SD + HFD *vs*. SD + HFD, including the cell types to which they are particularly linked and the biological processes to which they are applied **(C)**. The detailed DEGs for EC + SCD *vs*. SD + HFD are described **(D)**.

To identify neuroinflammatory changes associated with SD or an HFD in the brain, we employed a nanoString neuroinflammation panel (29), which covers 770 genes related to neuroinflammation in the brain. **Figure 4A** shows volcano plots of the DEGs between each group. Under the SCD, cathepsin S (*Ctss)*, endothelial cell adhesion molecule (*Esam*), and minichromosome maintenance complex component 6 (*Mcm6*) were the top three upregulated genes, and aspartate beta-hydroxylase (*Asph*), ribosomal protein S (*Rps21*), and BRCA-associated RING domain 1 (*Bard1*) were the top three downregulated genes after SD. The *mcm6*, C-C motif chemokine ligand 4 (*CCl4*), and interleukin 1 receptor kinase 3 (*Irak3*) genes were upregulated in the EC+HFD group compared to those in the EC+SCD group (**Figure 4A**).

Using DEGs from the four groups, we compared the expression patterns in each group. In the SD+HFD group, Fc epsilon receptor 1 g (*Fcer1g*), growth arrest and DNA damage inducible alpha (*Gadd45a*), and RAS like proto-oncogene B (*Ralb*) gene expression was significantly increased compared to that in the other groups (**Figure 4B**). **Figure 4C** shows the DEGs between each group and their roles in neuroinflammation. Under the SCD, five genes related to adaptive immune response, three genes related to microglial function, and three genes related to the cell cycle were differentially expressed after SD. Under the HFD, two genes related to cytokine signaling, two genes related to the innate immune response, and three genes related to microglial function were differentially expressed after SD. Adaptive immune response-related genes did not show significant differences between the SD+HFD and EC+HFD groups. Sialic acid-binding Ig-like lectin 1 (*Siglec1*), a marker for active neuroinflammation (30), was highly expressed in the SD+HFD group compared to that in the SD+SCD group. Compared with that in the EC+SCD group, the SD+HFD group had the highest number of DEGs (**Figure 4A**). These genes were related to inflammation, neuropathology, and microglial function (**Figure 4D**).

### 3.5 Integration analysis of gut microbiome and host gene expression

**Figure 5:**
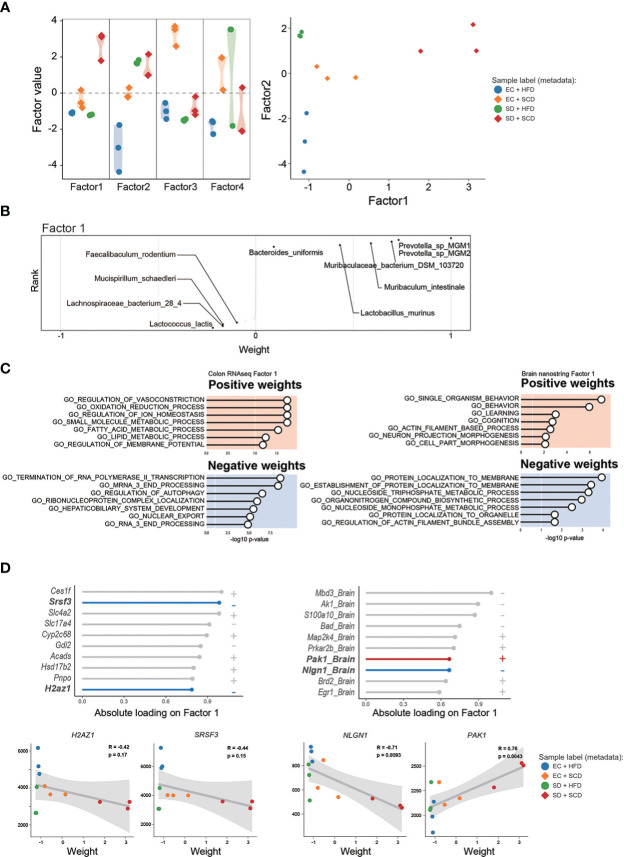
Multi-omics factor analysis from the gut microbiome to the colon and brain axis. The sample variation was represented by four captured factors of the multi-omics factor analysis (MOFA)2 and the distribution of the sample was plotted for factors 1 and 2 **(A)**. The weighted gut microbiome species in factor 1 are described following the ranks, and strongly associated species are described **(B)**. The seven significant gene sets related to Factor 1 are listed with *P*-values **(C)**. The top 10 features of factor 1 weights, which are estimated to provide a strong effect in specific variation, in colon RNA sequencing (left) and brain nanostring (right) are shown as weight values, and the correlation analysis **(D)**.

To identify the main factors that mediate the microbiota-gut-brain interactions, we performed multi-omics factor analysis (MOFA) by integrating microbiome and gene expression data (31, 32). **Figure 5A** displays the four determinants discovered by the factor analysis. Among the four factors, factors 1 and 2 showed effective discriminating values (**Figure 5A**; **Supplementary Figure 4**). The variable with the largest weight in factors 1 and 2 was from the microbiome layer. *Prevotella* sp. MGM1 in factor 1 and *Bacteroides satori* in factor 2 showed the highest weight in this analysis (**Figure 5B**; **Supplementary Figure 5**).

**Figure 5C** shows the GO terms with a high weight of factor 1 in the DEGs for the gene expression data. In the colon RNA sequencing data, “regulation of vasoconstriction,” “oxidation and reduction process,” “small molecule metabolic process,” and “Termination of RNA polymerase II transcription” were the top enriched GO terms. **Figure 5D** shows the absolute loadings of the top features of factor 1. Factor 1 was positively correlated with the P21(RAC1) activated kinase 1 (*Pak1*) gene in the brain and negatively aligned with Neuroglin 1 (*Nlgn1*) in the brain, as well as with serin/arginine-rich splicing factor 3 (*Srsf3*) and H2A.Z variant histone 1 (*H2az1*) genes in the colon (**Figure 5D**). Factor 2 was positively correlated with the Apoprotein E (*APOE*) and Calreticulin (*CALR*) genes in the brain and negatively aligned with the *Srsf3* and *H2az1* genes in the colon (**Supplementary Figure 5**).

## 4 Discussion

Here, we examined the effects of SD and diet-induced obesity on the gut microbiota, gut transcriptome, and brain gene expression. In addition, we integrated these data to reveal the main drivers of microbiota-gut-brain interactions. In the present study, we revealed the pleiotropic effects of SD and a HFD on the gut and brain. Previous studies have shown that SD can affect bodyweight by suppressing appetite (33) and decreasing energy expenditure (34). In both human and animal studies, SD have positive associations with obesity and weight gain (35, 36). However, our results did not show an effect of SD on body weight in either the standard chow diet (SCD) group or the HFD group, which may be due to the relatively short duration of SD in our experiment.

The HFD reduced gut microbiota biodiversity in terms of both alpha and beta diversity (**Figures 1C, D**). The gut transcriptome was primarily influenced by SD (**Figures 3A, B**), whereas brain gene expression associated with neuroinflammation was significantly altered following exposure to a HFD with SD (**Figures 4A, D**). Gut microbiota analysis revealed that the HFD caused dramatic changes in the physiology of the gut microbiota (**Figures 1**, **2**). SD did not induce robust changes in the gut microbiota, as in other previous studies (37, 38). Our results showed that the HFD both alone and with SD increased *Firmicutes* (**Table 2**), which has already shown significant correlations with obesity and sleep quality (39, 40).

Interestingly, the HFD alone increased the F/B ratio, whereas the HFD with SD decreased the F/B ratio by increasing *Bacteroidetes* (**Table 2**). Recent research by Gregory et al. has revealed a link between poor sleep and a higher body mass index (BMI), as well as a positive relationship between sleep quality and the F/B ratio (41). This is consistent with our findings, which show that sleep deprivation leads to a decrease in the F/B ratio. Recent findings also suggest that an increased F/B ratio leads to more effective glucose fermentation and higher nutritional absorption (42). This means that the increased F/B ratio in the HFD mice may be a compensatory reaction to overeating, and sleep disrupts this compensation. *Prevotella* species have shown negative associations with a HFD in mice (43) and significant relationships with weight change in a human randomized controlled trial (44). In this study, *Prevotella* sp. MGM1 and MGM2 levels were decreased in both the EC+HFD and SD+HFD groups compared to those in the other groups (**Figure 2B**).

The abundance of *Prevotella* and *Muribaculum* was higher in the SD+SCD group than that in the EC+SCD group but did not increase in the SD+HFD and EC+HFP groups. Recently, Badran et al. (45) reported that fecal microbiota transplantation using a fecal slurry, which has abundant levels of *Prevotella* and Muribaculaceae, improves sleep disturbances in mice. The composition of gut microbiota may adapt to defend against the stressors associated with sleep deprivation, while a high-fat diet (HFD) may complicate these protective responses. The gut microbiota is a critical factor in the body’s ability to adapt to stress, affecting neuroendocrine substances such as ghrelin and serotonin (46). This suggests that the gut microbiota plays a crucial role in regulating the body’s stress response. There is increasing evidence that gut microbiota may have a role in mitigating the effects of sleep disturbance. Studies in both humans and animals have shown that probiotic intervention can improve sleep quality (47, 48). To identify the possible mediators of microbiota-GBA interactions, we performed a microbial pathway analysis and found that the abundance of 5’-IMP synthesis-related species was highly increased in the SD+HFD group compared to that in the other groups (**Figure 2D**). 5’-IMP plays a key role in purine nucleotide synthesis and regulates various immune responses (49). In addition, 5’-IMP is important for hypnotic action in the brain (50). The adenosine and its metabolite inosine have been shown to be closely linked to the regulation of the circadian rhythm (51). Studies have shown that inosine activates the adenosine A2A receptor (52, 53), which plays a role in regulating sleep. Furthermore, research in animal models has demonstrated that inosine administration can increase neuronal proliferation in the brain and prevent depression-like behavior. Additionally, inosine has been found to prevent memory impairment in a rat model of Alzheimer’s disease (54).Therefore, increased 5’-IMP synthesis might be a protective response in the gut against metabolic stresses induced by SD and an HFD.

Gut transcriptome analysis revealed that SD caused robust changes in the gut transcriptome (**Figure 3**). In this study, the most significantly enriched pathways were related to ribosome biogenesis and nucleic acid metabolic processes (**Figure 3C**), which are critical for gut mucosal defense (55) and colorectal cancer progression (56). This result elucidates the current association between sleep, obesity, and colon cancer. Both SD and obesity are high-risk factors for colorectal cancer development (57, 58). Recent studies have shown deleterious effects of SD on the gut (14, 59). For example, SD causes premature death by increasing reactive oxygen species in the gut (14). We also found that SD enhanced the renin-angiotensin system (RAS)-associated pathways in the gut (**Figure 3C**). The gut RAS interacts bidirectionally with the gut microbiota and can promote intestinal inflammation and fibrosis (60, 61).

In the brain, genes related to neuroinflammation were altered by both SD and the HFD. The *Mcm6* gene was highly upregulated after SD under the SCD and HFD conditions (**Figure 4A**). This gene encodes a protein that is a component of the MCM complex, which is required for the initiation of eukaryotic genome replication (62). Furthermore, this gene has shown positive correlations with poor prognosis in brain and gastrointestinal tumors (63, 64). The most severe neuroinflammatory changes were observed in the SD+HFD group compared to those in the EC+SCD group. CXC motif chemokine ligand 10 (*Cxcl10*), insulin-like growth factor-1 (*Igf1*), and cluster of differentiation 70 (*Cd70*) were the top three highly elevated genes (Log2FC). These genes are well-known markers of proinflammatory signals (65–67).

To determine the main driver of the microbiota-gut-brain interactions, we performed factor analysis. MOFA2 revealed that the major feature of factors 1 and 2 was gut bacteria (**Figure 5**; **Supplementary Figure 5**). This suggests that the main driver of microbiota-gut-brain interactions with SD and an HFD is the gut microbiome. Notably, the *SRSF3* genes in the gut showed significant negative correlations with factors 1 and 2. According to recent studies, SRSF3 suppresses tumorigenesis (68) and inhibits cellular senescence (69). Thus, reduced *SRSF3* expression might be an important contributor to gastrointestinal dysfunction caused by SD and an HFD.

Our study had several limitations. First, we performed an MOFA based on microbiome and transcriptome data. Further evidence, such as the blood metabolome and gut proteome, is required to substantiate our conclusions. Second, we only tested adult male mice. As a result, sex differences and aging were not reflected in our study.

In summary, our study revealed novel associations between the gut microbiota and host responses after SD and diet-induced obesity. Obesity with SD has deleterious effects on gut and brain health. We discovered that the gut microbiota may be the primary driver of microbiota-gut-brain interactions, and 5’-IMP may be an essential microbial metabolite that facilitates gut-brain communication. These findings imply that healing gut dysbiosis may be a viable therapeutic target for enhancing sleep quality and curing obesity-related dysfunction.

## Data availability statement

The datasets presented in this study can be found in online repositories. The names of the repository/repositories and accession number(s) can be found in the article/**Supplementary Material**.

## Ethics statement

The animal study was reviewed and approved by the Institutional Animal Care and Use Committee of Gwangju Institute of Science and Technology (Approval number: GIST-2021-064).

## Author contributions

SL, C-MO, and TK contributed to the conceptual design of the project and the experiments described in the manuscript. The experiments were performed by JL and JK. The data were analyzed by JL, JK, and YK. The manuscript was written by JL, JK, and C-MO. Then, the manuscript was edited and critically evaluated by SL, C-MO, and TK. All authors contributed to the article and approved the submitted version.
