# Supplementary material for: Integrated analysis of the microbiota-gut-brain axis in response to sleep deprivation and diet-induced obesity
Source: Front Endocrinol (Lausanne). 2023 Feb 21;14:1117259. doi: 10.3389/fendo.2023.1117259 (PMC9990496; doi:10.3389/fendo.2023.1117259)

Supplementary Material

**Integrated analysis of the microbiota-gut-brain axis in response to sleep deprivation in a diet-induced obesity mouse model**

**Jibeom Lee^1, †^, Jiseung Kang^1, †^, Yumin Kim^1^, Sunjae Lee^2, *^, Chang-Myung Oh^1,*^, Tae Kim^1,*^**

^1^Department of Biomedical Science and Engineering, Gwangju Institute of Science and Technology, Gwangju 61005, Korea

^2^Department of School of Life Sciences, Gwangju Institute of Science and Technology, Gwangju 61005, Korea

† These authors contributed equally to this work.

*** Correspondence:**

Sunjae Lee, Ph.D.

E-mail: leesunjae@gist.ac.kr

Chang-Myung Oh, M.D, Ph.D.

E-mail: cmoh@gist.ac.kr

Tae Kim, M.D., Ph.D.

E-mail: [tae-kim@gist.ac.kr](mailto:tae-kim@gist.ac.kr)

**Supplementary Figure 1.** Body weight gain of mice after 8 weeks of standard chow diet or high fat diet.


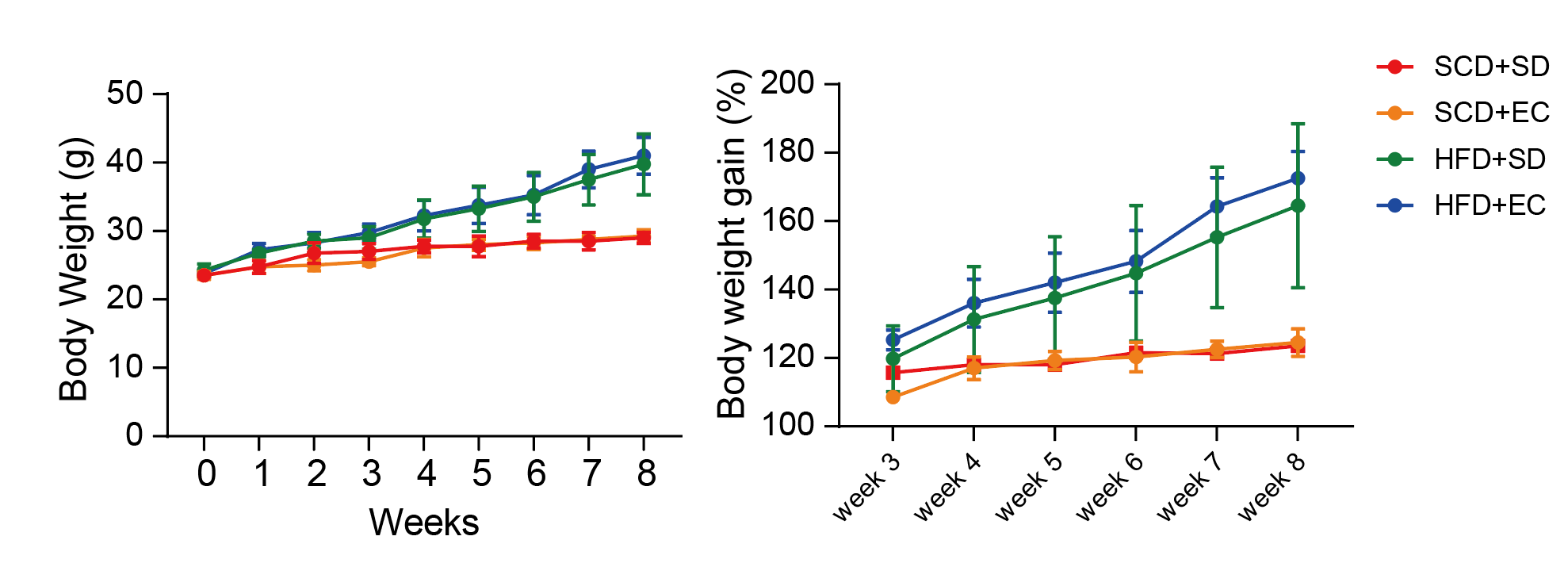


**Supplementary Figure 2.** Beta diversity, Bray-Curtis, and unweighted and weighted Unifrac.


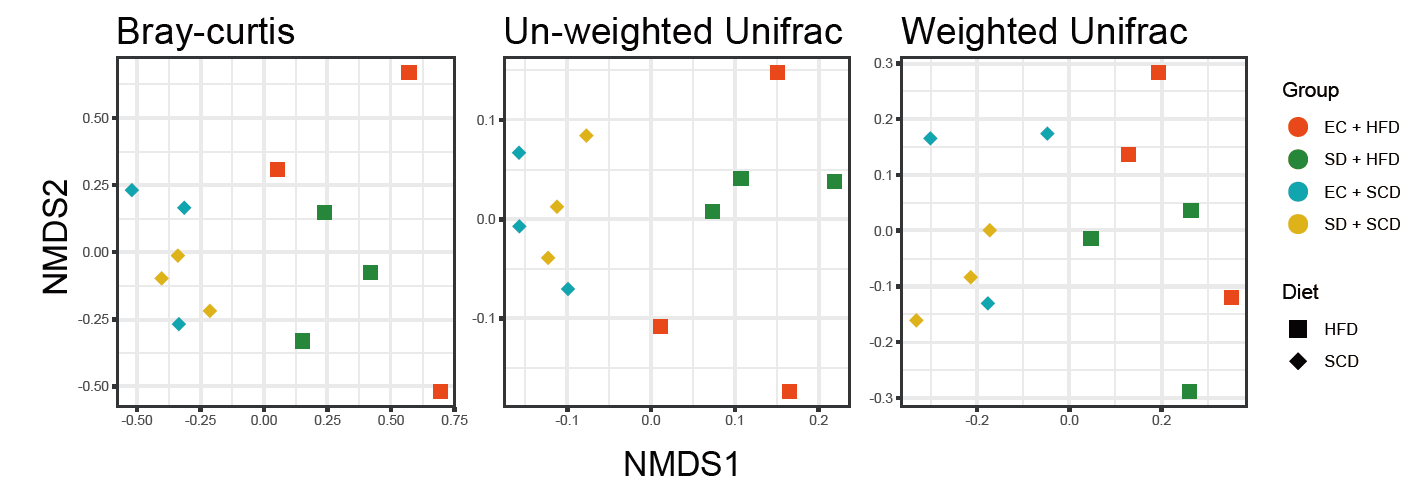


**Supplementary Figure 3.** Venn diagram for colon differentially expressed genes for sleep deprivation (SD) vs. exercise control (EC) under standard chow diet (SCD) and SD vs. EC under high fat diet (HFD) and their three intersecting genes.


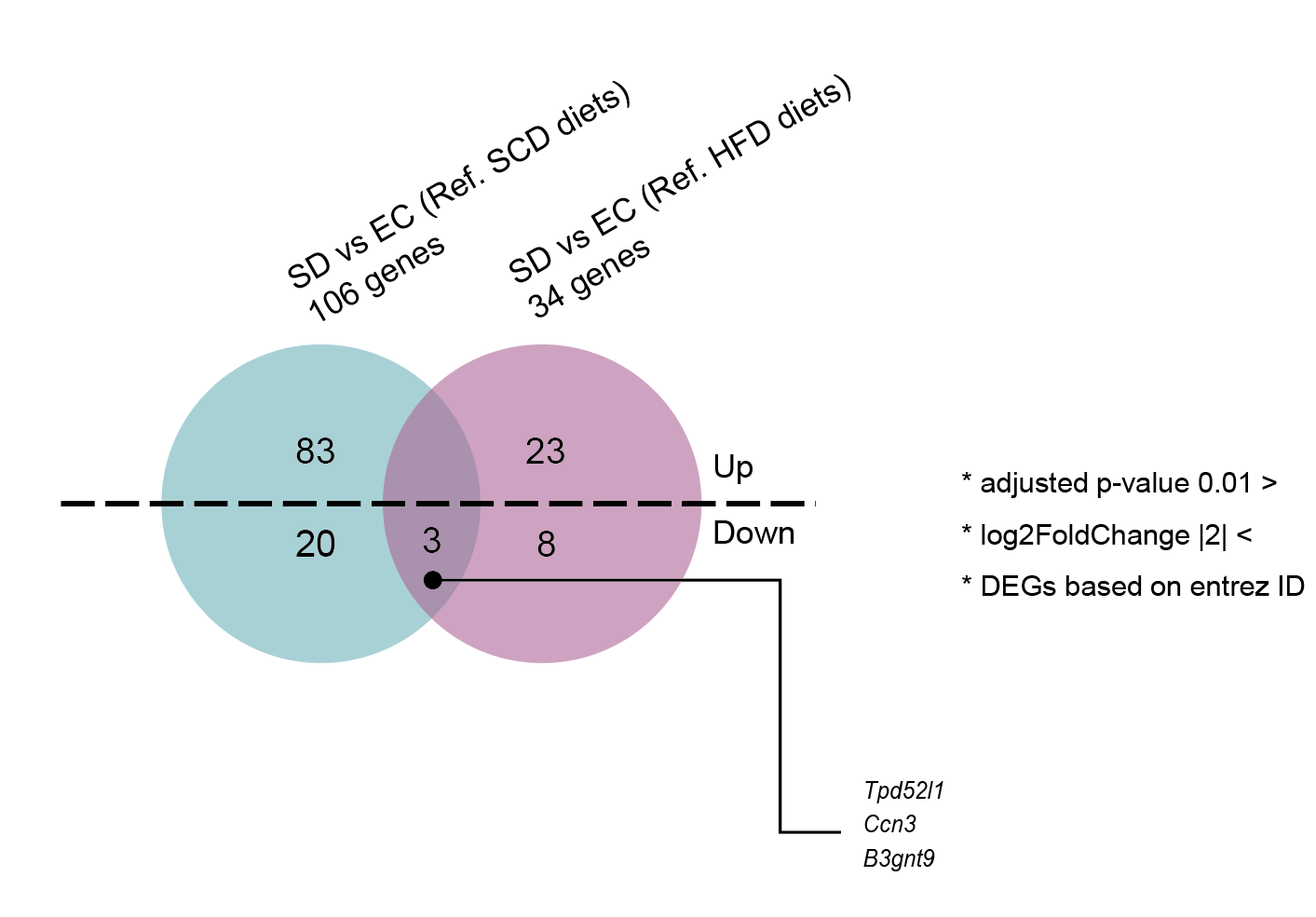


**Supplementary Figure 4.** An overview of variance composition of the multi-omics factor analysis for four datasets, colon RNA sequencing, Brain nCounter, microbiota phylogeny, and estimated gut microbiota genes. The barplot quantifies a total explanation for four factors.


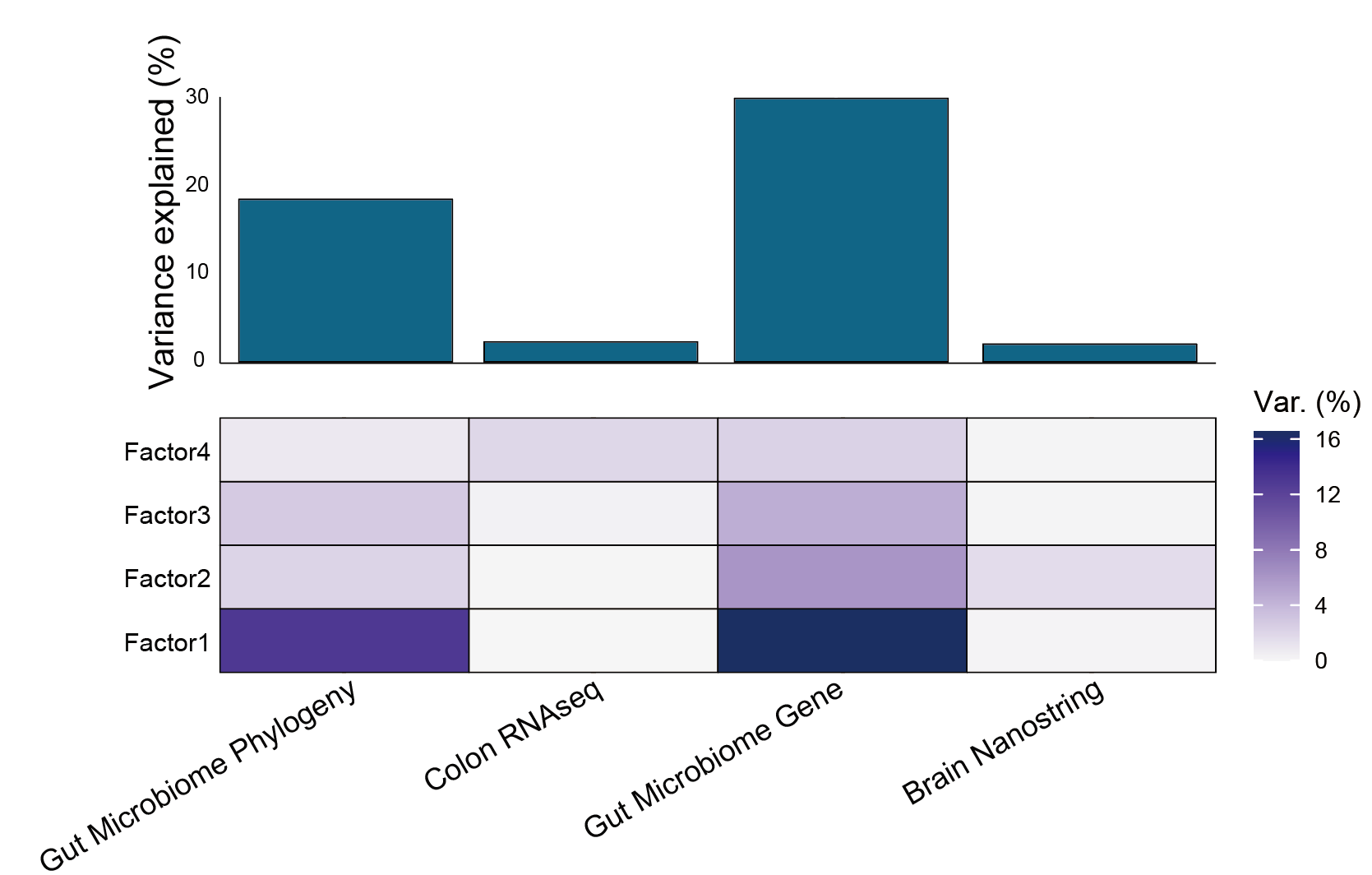


**Supplementary Figure 5.** The results of multi-omics factor analysis for factor 2.


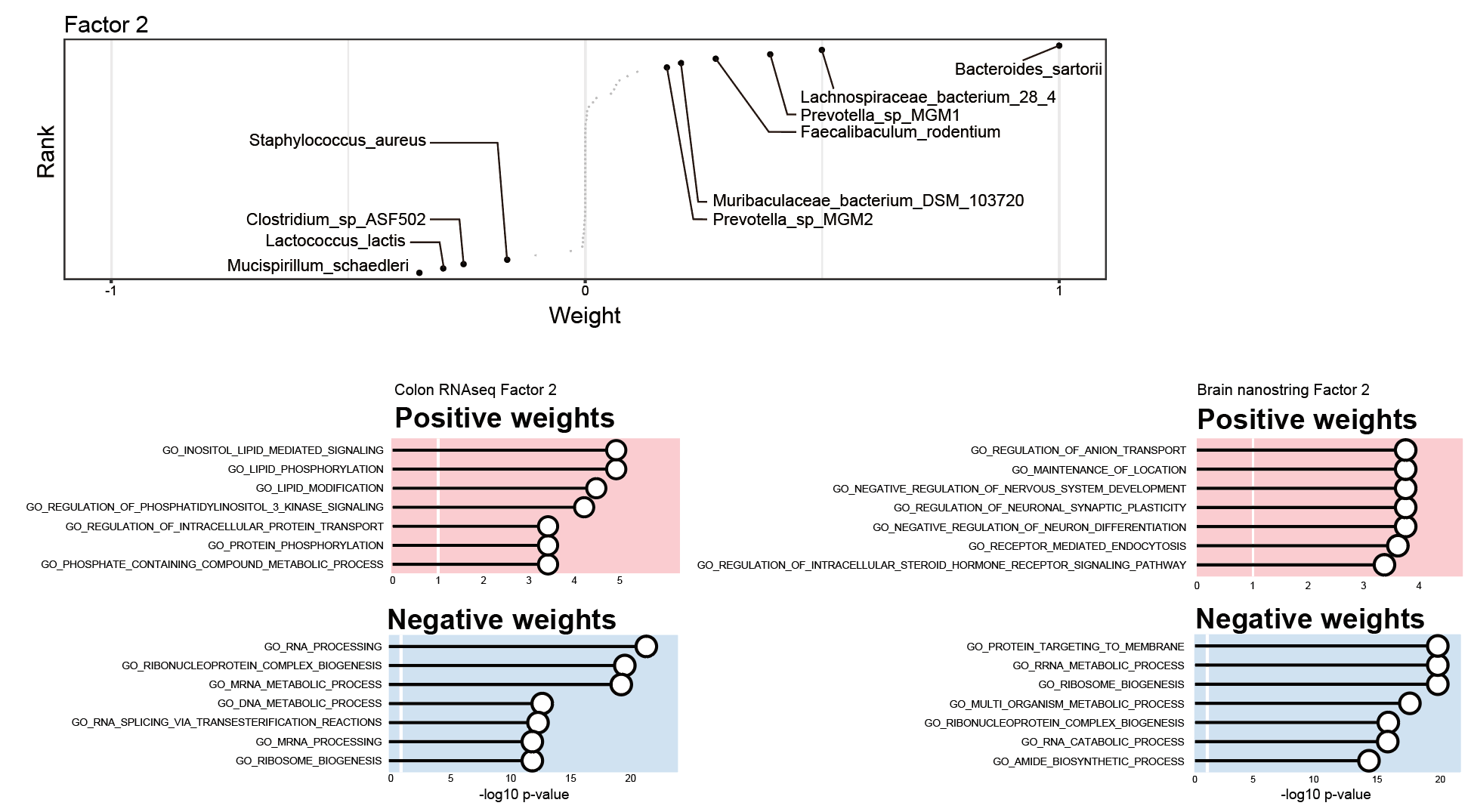


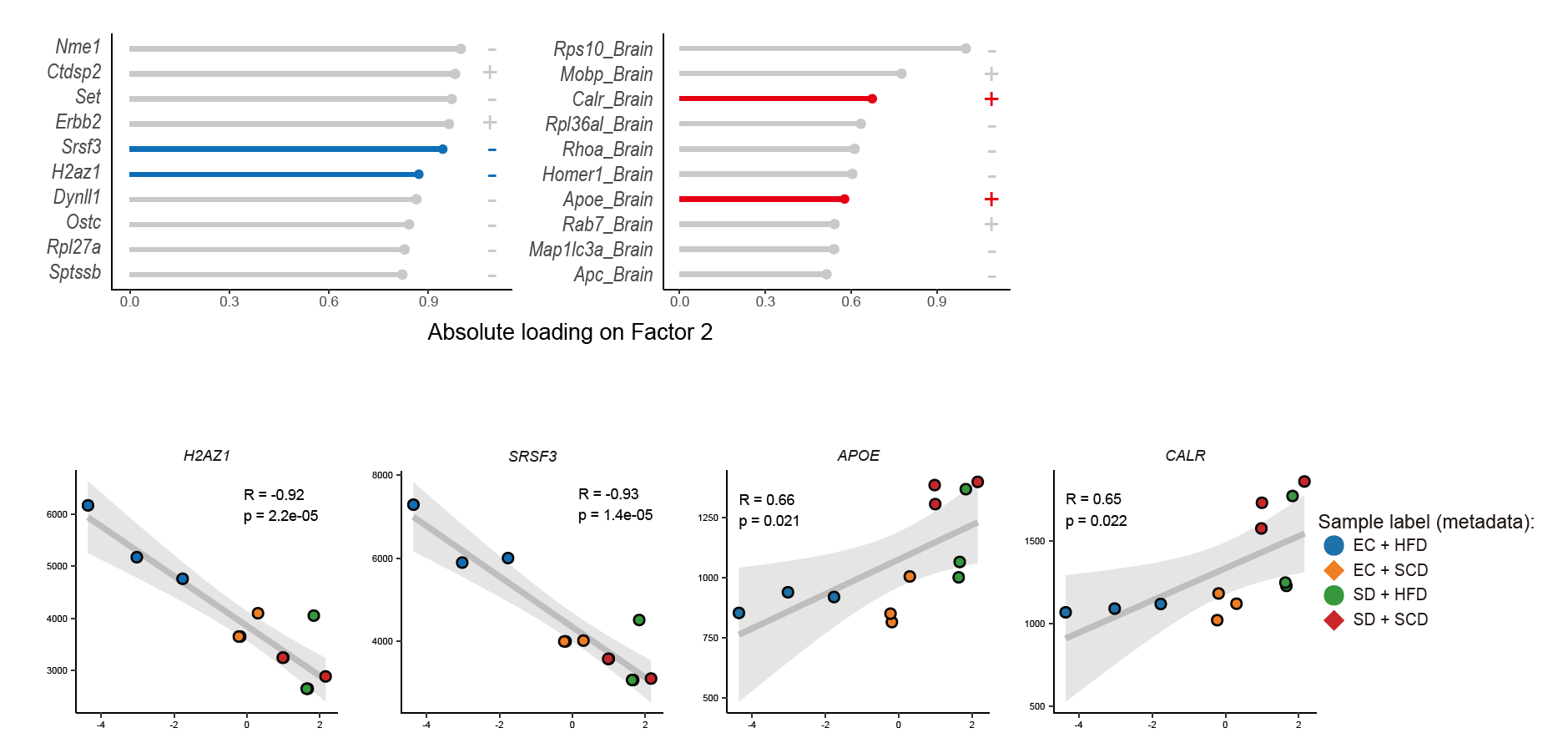

Supplement: Supplementary file 1 [file DataSheet_1.docx]
